# Influenza A(H3N2) Subclade K: Epidemiology, Molecular Evolution and Vaccine Effectiveness in Europe

**DOI:** 10.3390/pathogens15050474

**Published:** 2026-04-28

**Authors:** Irene Scarvaglieri, Maria Antonia De Francesco, Maria Alberti, Federico Cesanelli, Martina Salvi, Giorgio Tiecco, Francesco Castelli, Eugenia Quiros-Roldan

**Affiliations:** 1 Unit of Infectious and Tropical Diseases, Department of Clinical and Experimental Sciences, University of Brescia and ASST Spedali Civili di Brescia, 25123 Brescia, Italy; i.scarvaglieri@unibs.it (I.S.); m.alberti035@studenti.unibs.it (M.A.); f.cesanelli@unibs.it (F.C.); m.salvi026@unibs.it (M.S.); g.tiecco@unibs.it (G.T.); francesco.castelli@unibs.it (F.C.); 2 Highly Specialized Laboratory, ASST Spedali Civili of Brescia, 25123 Brescia, Italy; maria.defrancesco@unibs.it; 3 Institute of Microbiology, Department of Molecular and Translational Medicine, University of Brescia, 25123 Brescia, Italy; 4 Department of Global Public Health, Karolinska Institutet, 171 76 Stockholm, Sweden

**Keywords:** influenza A(H3N2) virus, subclade K, antigenic drift, vaccine effectiveness

## Abstract

Background: Influenza A(H3N2) viruses remain a major public health concern due to their rapid antigenic evolution and association with severe disease, particularly among high-risk populations. During the 2025–2026 influenza season, a marked epidemiological shift was observed in Europe, with the emergence and predominance of the A(H3N2) subclade K (J.2.4.1). Objectives: This narrative review aims to provide an integrated overview of the epidemiology, evolutionary dynamics, and public health implications of subclade K, with a particular focus on its impact on vaccine effectiveness, in comparison with the 2024–2025 influenza season. Methods: A non-systematic literature review was conducted using major scientific databases and official public health sources, including WHO and ECDC reports. Recent surveillance data, genomic analyses, and epidemiological updates were included. Given the rapidly evolving evidence base, selected preprint studies were also considered and interpreted with caution. Results: The 2025–2026 influenza season in Europe was characterized by a relative genetic convergence, with subclade K accounting for the majority of A(H3N2) sequences. This variant demonstrated a clear selective advantage and was associated with an earlier and more intense epidemic peak. Molecular analyses indicate the accumulation of multiple mutations in the hemagglutinin protein, particularly within key antigenic sites, contributing to immune escape. These evolutionary changes have important implications for vaccine effectiveness, with current estimates suggesting moderate protection against infection but preserved effectiveness against severe outcomes. Antigenic mismatch, manufacturing constraints, and host-related factors further contribute to reduced vaccine performance. Conclusions: The emergence and rapid spread of subclade K highlight the dynamic nature of influenza virus evolution and its impact on public health. Continuous genomic surveillance and timely vaccine updates remain essential. Despite suboptimal effectiveness against infection, influenza vaccination continues to provide significant protection against severe disease and should remain a cornerstone of prevention strategies.

## 1. Background

Influenza A(H3N2) viruses continue to represent a major public health concern due to their rapid antigenic evolution and their association with severe disease, particularly among vulnerable populations [1]. During the 2025–2026 influenza season, epidemiological data indicate a marked shift from a heterogeneous pattern of co-circulating influenza viruses to a relative genetic convergence dominated by A(H3N2) subclade K, which accounted for the majority of sequenced strains across Europe [2,3]. Despite regional variability, this lineage demonstrated a clear selective advantage and was associated with an earlier and more intense seasonal peak [2].

This narrative review aims to provide an integrated overview of the epidemiology, molecular characteristics, and public health implications of subclade K with a focus on vaccine effectiveness, including a comparison to the 2024–2025 season.

## 2. Methods

This narrative review was conducted to provide a comprehensive and up-to-date overview of the epidemiology, evolutionary dynamics, and public health implications of influenza A(H3N2) subclade K. A non-systematic literature search was performed using electronic databases including PubMed, Scopus, and Google Scholar, as well as official reports from international public health agencies such as the World Health Organization (WHO) and the European Centre for Disease Prevention and Control (ECDC).

The search strategy combined keywords related to influenza A(H3N2), including “H3N2”, “subclade K”, “J.2.4.1”, “antigenic drift”, “vaccine effectiveness”, and “influenza epidemiology”, focusing primarily on studies published between 2023 and 2026. Additional sources included surveillance reports, genomic summaries, and epidemiological updates from national influenza centres and the Global Influenza Surveillance and Response System (GISRS).

Studies were selected based on predefined relevance criteria, including: (i) direct focus on influenza A(H3N2) subclade K or closely related lineages; (ii) provision of epidemiological, genomic, or vaccine effectiveness data; and (iii) relevance to the European context or comparison between the 2024–2025 and 2025–2026 seasons.

Priority was given to peer-reviewed studies and official surveillance reports. Nevertheless, given the rapidly evolving nature of the topic, preprint articles were also considered when relevant to emerging data on subclade K; however, these findings were interpreted with caution, clearly identified as non-peer-reviewed evidence and their findings were interpreted with caution and not used as sole support for key conclusions.

In the absence of formal systematic review methods, study selection and synthesis were guided by the aim of providing a comprehensive and up-to-date narrative overview, rather than exhaustive coverage.

## 3. Epidemiology

Influenza A(H3N2) emerged in 1968 as a result of an antigenic shift driven by reassortment between an avian influenza A virus and the human A(H2N2) strain that had been circulating since 1957. The pandemic virus, A/Hong Kong/1968 (H3N2), acquired two gene segments from a low-pathogenic avian virus—including the hemagglutinin (H3)—while retaining six internal genes from the human-adapted H2N2 virus [4]. The 1968 flu pandemic resulted in an estimated one million to four million deaths worldwide [5].

Further, this subtype has long been recognised as one of the most clinically significant influenza subtypes, largely due to its pronounced capacity for antigenic drift and its disproportionate impact on older adults and medically vulnerable individuals [1,6]. This evolutionary plasticity is primarily driven by the accumulation of mutations in the hemagglutinin (HA) protein, the principal antigenic determinant targeted by neutralising antibodies, allowing the virus to evade pre-existing immunity at the population level [7]. Within this dynamic evolutionary landscape, the emergence of J.2.4.1—more commonly named subclade K—represents a further step in the ongoing diversification of H3N2 viruses, with significant implications for both epidemiology and vaccine performance [8]. In Europe, the epidemiology of influenza A(H3N2) during recent seasons has been characterised by fluctuating dominance of distinct genetic subclades, reflecting continuous viral evolution under immune pressure [3]. The 2024–2025 season showed a heterogeneous pattern, with multiple co-circulating influenza virus types and subtypes contributing to a complex epidemiological scenario [9]. During the reporting period, in Europe a total of 354,455 influenza virus detections were recorded, of which 73% were type A and 26% type B, with a small proportion remaining untyped [10]. Among subtyped influenza A viruses, A(H1N1)pdm09 predominated, accounting for 60% of cases, while A(H3N2) represented 40%, indicating substantial co-circulation of the two major influenza A subtypes [10]. In parallel, influenza B viruses contributed significantly to overall circulation, representing more than one-quarter of detections, with almost all characterised strains belonging to the B/Victoria lineage [10].

**Figure 1:**
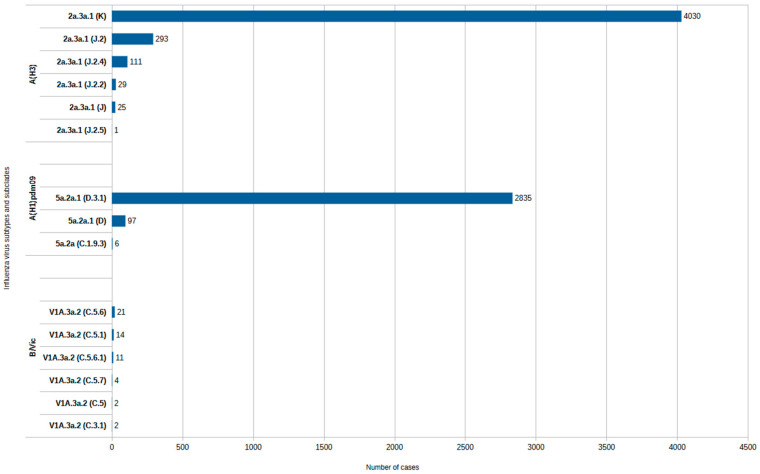
Distribution of A/H3N2 subtypes and subclades in Europe during 2025–2026 influenza season according to Erviss database.

In contrast, the 2025–2026 season has been marked by a relative genetic convergence, with subclade K (J.2.4.1) rapidly becoming the predominant circulating lineage across multiple European countries [3]. Across participating European countries, influenza A viruses accounted for the majority of detected influenza cases (>70–80% in sentinel surveillance), with A(H3N2) representing the dominant subtype (~85%) [3]. According to the European Respiratory Virus Surveillance Summary, subclade K accounted for 89% of the A(H3) virus sequences collected by National influenza centres from all EU/EEA countries between 1 October 2025 and 15 March 2026 [11]. In Figure 1 we show the distribution of A/H3N2 subtypes and subclades in Europe during 2025–2026 influenza season.

Country-specific analyses further highlight important epidemiological nuances across Europe. According to national surveillance data, in both Italy and France, a pattern of sustained co-circulation between A(H3N2) and A(H1N1)pdm09 was observed. In Italy, influenza A viruses were overwhelmingly predominant (99.6%), with A(H3N2) accounting for 62.6% of subtype strains and A(H1N1)pdm09 for 37.4%, indicating a substantial contribution of both subtypes throughout the season. Sequencing data showed that most A(H3N2) viruses belonged to subclade K [12]. Similarly, in France, A(H3N2) represented 59% of detected influenza viruses compared with 36% A(H1N1)pdm09, again reflecting notable co-circulation [13]. Importantly, early-season data indicated an initial predominance of A(H1N1)pdm09, followed by a progressive shift towards A(H3N2) dominance as the epidemic evolved, with subclade K being the most frequently detected among A(H3N2) viruses [13].

In contrast, a more marked predominance of A(H3N2) was observed in England. Between week 40 in 2025 and week 12 in 2026, 1582 out of 1839 genetically characterised influenza viruses (86%) were A(H3N2), compared with only 13% A(H1N1)pdm09 and 1% influenza B, indicating a substantially stronger dominance of H3N2 compared with other European settings [14]. This shift toward dominance of a single subclade suggests a selective advantage, consistent with epidemiological observations from Australia, the United Kingdom, Japan, and Europe indicating that the subclade K has increased transmission fitness and the ability to sustain prolonged seasonal activity [15]. The temporal pattern of influenza activity during the 2025–2026 season has remained broadly consistent with the typical winter peak observed in temperate regions, although ECDC surveillance data showed an earlier than expected increasing trend, occurring three to four weeks earlier than the two most recent seasons, and at the earliest range of five pre-COVID-19 pandemic seasons [2]. Current data do not indicate a marked increase in disease severity at the individual level compared to previously circulating H3N2 strains [16]. Nevertheless, the overall burden on healthcare systems has been substantial due to the higher number of infections, particularly among high-risk populations [17]. Importantly, infections caused by H3N2 viruses have been consistently associated with higher rates of hospitalisation and mortality compared to other influenza subtypes, reinforcing the clinical relevance of monitoring emerging variants such as subclade K [15]. Consistent with historical patterns of H3N2 infection, the groups most at risk of severe disease include older adults, young children, pregnant women, and individuals with chronic co-morbidities such as cardiovascular, respiratory, and metabolic diseases [6,17]. Immunocompromised individuals also represent a particularly vulnerable group due to impaired immune responses, which limit both natural and vaccine-induced protection [6].

## 4. Evolutionary Dynamics of Influenza Viruses

Influenza viruses belong to the family *Orthomyxoviridae* and they are characterised by having a single-stranded, negative sense, and segmented RNA genome [18]. Their evolution is driven by two main mechanisms: antigenic drift and antigenic shift [19]. Antigenic drift refers to the slow accumulation of point mutations in the major outer glycoproteins—hemagglutinin and neuraminidase (NA). In contrast, antigenic shift involves the reassortment of genetic segments between different influenza strains during co-infection [20,21]. Such genetic changes enable the viruses to escape immune recognition, creating ongoing challenges and requiring continual updates to influenza vaccines. Mutations in the HA gene play a pivotal role in antigenic changes because they affect the virus’s ability to bind to host cells, thereby facilitating entry and infection [22]. NA supports viral replication and release, and variations in this gene can also result in resistance to antiviral treatments [23]. Therefore, twice a year, a WHO-convened panel of experts issues recommendations for vaccine composition for both the Northern and Southern Hemispheres [24]. These decisions rely on genetic and antigenic data produced by WHO Influenza Collaborating Centers and National Influenza Centers (NICs), based on virus samples collected through the Global Influenza Surveillance and Response System (GISRS) network [25]. Conventional influenza vaccines target influenza A(H1N1)pdm09, A(H3N2), and influenza B virus [26].

**Figure 2:**
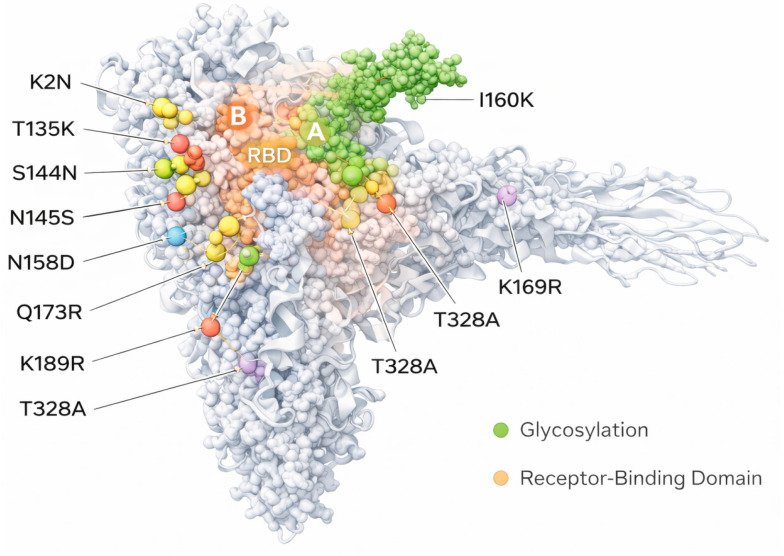
Amino acid substitutions in the hemagglutinin of Influenza A/H3N2 virus responsible for the majority of antigenic change.

Although the antigenic evolution of HA has been widely investigated, interest in NA antigenic mutations has only more recently increased because of their relevance for influenza vaccine efficacy [26]. It was observed that the antigenic changes of N2 NA progressed more slowly than that observed for H3 HA, and the antigenic mutations of NA and HA did not always align [26]. This asynchrony antigenic drift between the two proteins may improve seasonal influenza vaccine effectiveness, as a well-matched NA component could enhance protection even in seasons when the HA component is mismatched [26]. For the HA of A(H3N2) viruses, antigenic drift was shown to arise mainly from substitutions at seven amino acid positions near the receptor-binding site (RBS) [27]. Similar patterns have been identified for A(H5N1), A(H1N1), and influenza B virus HA [28,29]. This is likely because antibodies targeting the RBS are particularly effective at neutralising the virus; therefore, mutations at these epitopes most readily enable immune escape in humans [26]. In contrast, NA antigenic drift involved substitutions at numerous amino acid sites. The most crucial changes occurred close to the catalytic site (positions 197, 199, 221, 245, 247, 248, 346, and 369), at the top of the protein where NA monomers interact (positions 143 and 468), and along the lateral surface of the NA tetramer (positions 216, 249, 253, 313, 329, 336, 338, and 401) [26]. Because some antibodies inhibit NA activity by blocking its active site, mutations at these positions can directly alter antibody binding [26]. Based on preliminary data from a preprint study, the genetic drift of H3N2 viruses from related J.2.4 viruses has led to the recent emergence of the subclade K variant with an accumulation of mutations in the HA protein that alter antigenic properties [30]. Notably, recent H3N2 variants such as J.2.3 and J.2.4 have acquired substitutions at critical antigenic positions, suggesting an accelerated evolutionary trajectory [30]. A total of 10 amino acid substitutions (K2N, T135K, S144N, N145S, N158D, I160K, Q173R, K189R, T328A and S378N) were identified within antigenic sites of HA of Influenza A(H3N2) viruses when compared to the Northern Hemisphere 2025–2026 H3N2 vaccine strain (Figure 2) [31]. Eight of these substitutions occurred in the HA1 subunit, seven of which mapped to known antigenic sites A, B, and D and the receptor-binding domain (RBD) [31].

These observed mutations are more numerous than those reported in previous seasonal evolutionary patterns and are associated with conformational alterations in the hemagglutinin (HA) structure, thereby reducing the binding affinity of neutralising antibodies elicited by prior infection or vaccination [32]. In addition to direct amino acid substitutions, changes in glycosylation patterns have also been observed, with the addition of glycan shields that mask antigenic epitopes and further contribute to immune evasion [15]. Such mechanisms are well recognised in H3N2 evolution and represent a major challenge for maintaining effective population immunity [8]. The comparison between the 2024–2025 and 2025–2026 seasons highlights the dynamic nature of influenza virus evolution and its impact on public health [3]. During the earlier season, the presence of multiple co-circulating variants resulted in a broader antigenic landscape, whereas the subsequent dominance of subclade K reflects a process of selective sweep driven by immune pressure [8].

## 5. Vaccine Effectiveness

This evolutionary shift, based on preliminary data from a preprint study, may have important implications for vaccine effectiveness, as antigenic drift can allow them to partially evade host immunity associated with previous vaccination or infection [33]. Indeed, the emergence of subclade K occurred after the selection of vaccine components, limiting the ability of the vaccine to provide optimal protection [17].

Current estimates suggest moderate effectiveness against infection, with considerable variability depending on age group, study design, and clinical setting [3]. In particular, recent data from an English cohort indicate that vaccine effectiveness against laboratory-confirmed A(H3N2) infection ranged approximately between 20% and 40% in adults, with somewhat higher estimates in paediatric populations, depending on prior immunity and vaccine type [34]. In the same analysis, effectiveness against severe outcomes such as hospitalisation was higher, reaching values of approximately 40–60% in some cohorts [34], confirming a retained protective effect against severe outcomes, including hospitalisation and death, particularly among high-risk individuals [15]. This apparent discrepancy reflects the complex interplay between viral evolution and host immune responses, where partial immunity may still mitigate disease severity even when infection is not fully prevented [6]. Several factors contribute to the incomplete effectiveness of influenza vaccines against subclade K.

Preliminary evidence from preprint studies suggests that antigenic mismatch is a key contributing factor, resulting from the rapid evolution of the HA protein and the time lag inherent in vaccine production [35], and leading to lower neutralising antibody titres post-vaccination [36]. Additionally, the use of egg-based manufacturing processes can introduce adaptive mutations that alter the antigenic properties of the vaccine strain, further reducing its similarity to circulating viruses [17]. In this context, relatively limited changes in the hemagglutinin protein may substantially impair antibody recognition induced by prior infection or vaccination, with important implications for vaccine strain selection [37]. Notably, several studies have suggested improved antigenic fidelity and, in some cases, higher effectiveness for cell-based or recombinant vaccines compared with egg-based formulations, highlighting the impact of the production platform on vaccine performance [37].

Host-related factors, including age-related immune decline and prior exposure history, also play a significant role in shaping vaccine responses [6,38]. The concept of original antigenic sin (OAS) provides an additional explanatory framework highly relevant to ageing populations [15]. OAS refers to the propensity of the immune system to preferentially recall antibody responses elicited by the first influenza strains encountered early in life upon subsequent exposure to antigenically drifted viruses [39]. This results in the dominance of memory B cells targeting historical epitopes, while constraining the recruitment of naïve B cells capable of recognising newly emerged antigenic sites [15]. This long-lasting immune imprinting shapes influenza susceptibility and vaccine responsiveness across the life course. In older adults, whose primary exposures often involved antigenically distinct historical strains, immune responses may be biased toward obsolete epitopes, thereby limiting effective neutralisation of contemporary drift variants such as H3N2 K subclades [15]. In the context of substantial antigenic divergence, this effect may act synergistically with immunosenescence, further reducing vaccine-induced protection [15].

These combined factors explain why influenza vaccines rarely achieve complete protection, particularly against highly variable subtypes such as H3N2 [32]. Nevertheless, vaccination remains the most effective measure to prevent severe influenza outcomes. Even in seasons characterised by antigenic mismatch, vaccines are consistently associated with protection against severe disease, hospitalisation, and death [2]. Public health evidence further supports the importance of maintaining high vaccine uptake. Modelling studies conducted at the EU/EEA level have shown that higher influenza vaccination coverage, particularly among older adults, is strongly associated with a reduced disease burden [34]. This is especially relevant in the context of early and intense influenza seasons, where timely vaccination can mitigate pressure on healthcare systems and reduce transmission, including through indirect protection from vaccinated populations such as healthcare workers and children [34]. Despite these benefits, influenza vaccination coverage remains suboptimal across Europe. According to Eurostat data, only 47.1% of individuals aged ≥65 years received seasonal influenza vaccination in 2023, reflecting a concerning gap in protection among those at highest risk [40]. This trend may be partly linked to increased vaccine hesitancy observed in recent years, particularly following the SARS-CoV-2 pandemic [40]. Taken together, these findings underscore the critical need to strengthen vaccination strategies and public health messaging, particularly in seasons characterised by the emergence of antigenically drifted variants such as A(H3N2) subclade K.

In this setting, antiviral therapy represents an important complementary intervention, particularly for individuals at higher risk of severe disease [2]. Early initiation of antivirals is essential to reduce the likelihood of complications and disease progression, especially when vaccine effectiveness may be suboptimal due to antigenic mismatch [2].

From a public health perspective, the emergence of subclade K underscores the importance of continuous genomic and antigenic surveillance of influenza viruses [8]. Timely identification of emerging variants is essential for informing vaccine strain selection and guiding preventive strategies [3]. Recent recommendations from the European Medicines Agency for the 2026–2027 influenza season further highlight the need for continuous vaccine updates in response to ongoing antigenic drift. For egg-based quadrivalent vaccines, the European Medicines Agency (EMA) recommended inclusion of an A(H3N2) virus belonging to the A/Darwin/1454/2025 (H3N2)-like virus, replacing previously used strains to better match recently circulating variants. Similar updates were recommended for cell-based and recombinant vaccines, with strain selection aligned to the most antigenically relevant circulating A(H3N2) viruses [41]. These updates reflect the rapid evolution of A(H3N2), including the emergence of drifted variants such as subclade K, and underline the limitations of current strain prediction approaches. While seasonal vaccines remain essential to reduce severe disease, these challenges support the development of next-generation platforms, including cell-based, recombinant, and mRNA vaccines, as well as broader strategies targeting conserved viral regions [32]. Such advances may help overcome the limitations associated with current seasonal vaccines and reduce the impact of antigenic drift [32].

## 6. Future Perspectives and Limitations of the Study

Subclade K represents a significant example of ongoing influenza virus evolution, illustrating how antigenic drift can influence epidemiology, clinical outcomes, and vaccine performance.

Its rapid spread across Europe during the 2025–2026 season highlights the challenges faced in controlling H3N2 viruses and the need for adaptive and forward-looking public health strategies [3].

Although current vaccines provide only partial protection against infection, they remain a critical tool for reducing severe disease and should continue to be widely implemented [15].

The study presents several limitations. First, it incorporates evidence derived from preprint publications, which were included in light of the rapidly evolving and time-sensitive nature of the research topic. Second, the 2025–2026 influenza season in the Northern Hemisphere is still ongoing and has not yet been formally concluded; however, currently available surveillance data already suggest a clear decline in activity.

Future efforts should focus on enhancing surveillance systems and improving vaccine design to better anticipate and respond to emerging variants such as subclade K.

## Data Availability

No new data were created or analysed in this study. Data sharing is not applicable to this article.
